# Health research needs more comprehensive accessibility measures: integrating time and transport modes from open data

**DOI:** 10.1186/s12942-016-0052-x

**Published:** 2016-07-28

**Authors:** Henrikki Tenkanen, Perttu Saarsalmi, Olle Järv, Maria Salonen, Tuuli Toivonen

**Affiliations:** 1 Department of Geosciences and Geography, University of Helsinki, P.O. Box 64, Gustaf Hällströmin katu 2, 00014 Helsinki, Finland; 2 National Institute for Health and Welfare, P.O. Box 30, Mannerheimintie 166 A, 00271 Helsinki, Finland

**Keywords:** Accessibility, Health, Door-to-door approach, Spatio-temporal, Multimodal, Public transport, Private car, Open data, Travel time, Food

## Abstract

**Background:**

In this paper, we demonstrate why and how both temporality and multimodality should be integrated in health related studies that include accessibility perspective, in this case healthy food accessibility. We provide evidence regarding the importance of using multimodal spatio-temporal accessibility measures when conducting research in urban contexts and propose a methodological approach for integrating different travel modes and temporality to spatial accessibility analyses. We use the Helsinki metropolitan area (Finland) as our case study region to demonstrate the effects of temporality and modality on the results.

**Methods:**

Spatial analyses were carried out on 250 m statistical grid squares. We measured travel times between the home location of inhabitants and open grocery stores providing healthy food at 5 p.m., 10 p.m., and 1 a.m. using public transportation and private cars. We applied the so-called door-to-door approach for the travel time measurements to obtain more realistic and comparable results between travel modes. The analyses are based on open access data and publicly available open-source tools, thus similar analyses can be conducted in urban regions worldwide.

**Results:**

Our results show that both time and mode of transport have a prominent impact on the outcome of the analyses; thus, understanding the realities of accessibility in a city may be very different according to the setting of the analysis used. In terms of travel time, there is clear variation in the results at different times of the day. In terms of travel mode, our results show that when analyzed in a comparable manner, public transport can be an even faster mode than a private car to access healthy food, especially in central areas of the city where the service network is dense and public transportation system is effective.

**Conclusions:**

This study demonstrates that time and transport modes are essential components when modeling health-related accessibility in urban environments. Neglecting them from spatial analyses may lead to overly simplified or even erroneous images of the realities of accessibility. Hence, there is a risk that health related planning and decisions based on simplistic accessibility measures might cause unwanted outcomes in terms of inequality among different groups of people.

## Introduction

Human mobility is an indisputable component to the functioning of our societies [1]. From a social perspective, differentiation in the willingness and ability of individuals to move in physical space can be considered as a factor that contributes to social equality or inequality [2, 3]. Moreover, the interaction of an individual with the surrounding physical environment and social structures shapes not only one’s daily life practices [4], but also one’s health conditions [5–7]. Scholars from the disciplines of geography, urban planning, transportation, epidemiology and health research seek to better understand the linkage between individuals’ health and surrounding environment, such as socio-spatial inequalities in access to healthy food [8–12]. The discourse on access to food is largely about “food deserts”—a spatial concept referring to a geographical area where residents are lacking spatial and socio-economic access to affordable, healthy, and nutritious food [9, 13]. It is commonly considered that without access to healthy food, residents are left with an unhealthy diet leading to increased risk of obesity, diabetes, other chronic illnesses, and generally worsened health [8, 11, 14–20]. However, in analytical studies, the association between access to healthy food or “food deserts” and adverse health outcomes for people remain unclear [21–25]. One reason for the contradictory results may be the differences between conceptual approaches and methodological choices used to calculate accessibility.

Knowledge regarding the ability or need for people to move in order to access services, such as health services or healthy food, is commonly attained through the concept of accessibility [26], which can be defined as the ease of reaching a destination from a specific location using a particular transport mode [27]. Methodologically, in health related research spatial accessibility is measured using different metrics [28], which are predominantly either a naïve count of opportunities within a spatially fixed geographic unit (census tract, buffer zone) or a simple distance measure based on either a rough straight-line distance or road network metrics [11, 13, 19, 29–35]. Such metrics are easy to calculate and fairly robust, however they are static in nature and ignore the dynamics of people’s daily life. Moreover, conceptually speaking, studies neglect two essential domains in current (healthy) food accessibility research: temporality and multimodality. Temporality is a particularly important aspect in a 24/7 society, where people are living with increasingly individual daily rhythms [36]. Considering the mode of transportation, on the other hand, is crucial for considering accessibility across the broad social spectrum of society, including disadvantaged subgroups.

In this research, we aim to contribute to the theoretical and methodological framework of health related accessibility research. We demonstrate the importance of integrating various modes of transportation as well as temporal dynamics in transport and service networks to accessibility analyses. We test and apply this approach to study healthy food accessibility in an urban context. We propose an advanced framework for measuring spatio-temporal food accessibility by transport mode, and present: (1) how spatial access to healthy food is influenced by transport mode in time, and (2) how accessibility patterns vary spatially and temporally in relation to population distribution. The proposed framework provides more realistic analyses of food accessibility, which could help to better understand the association between accessibility and health.

## Background

### Human mobility and temporality of food accessibility

In line with calls emphasizing temporality and a person-based approach in social sciences [7, 37], there is a trend of incorporating temporal perspectives [38, 39] and a person-based approach [25] in food accessibility research, and measuring access to food beyond residential locations [40]. While spatial accessibility consists of temporal, physical, organizational, and financial components [26], the temporal dimension is essential from three different aspects.

First, arguably the measure closest to an individual’s embodied experience is that of travel time (or travel speed) instead of merely travelled distance, per se, due to the cognition of and/or the valuation of travel time [41]. Two other time-dependent aspects are demand and supply sides of access to and the use of healthy food opportunities by people.

From the demand side, time to access and buy healthy food depends on the persons’ complex spatial activity-travel behavior that varies on an hourly, daily, and weekly basis [41], and is influenced by seasonality [42, 43]. In our contemporary 24/7 society, activity-travel behavior of people is becoming more flexible and fragmented in space and time [44], particularly due to the flexibility in working schedules and night-shift working [45]. Thus, food purchasing is occurring throughout the day and includes a need for night shopping [46]. Neglecting the latter in calculating access to healthy food may lead to biased input regarding accessibility measures for health studies.

From the supply side, temporal variations in healthy food accessibility depends on both the structure of the facilities providing food as well as transport system. Opening hours of facilities providing food determine access to food as beyond opening hours the desired services are still inaccessible, regardless of spatial location [47, 48]. Access to food also changes on a monthly basis (seasonal markets) and across years as the network of facilities providing food changes [32, 49–51]. In the case of transport systems, access to food stores by public transport depends on time schedules, frequencies and transfer times of public transport and considering time-of-day, weekday and seasonality [52]. Even though users of private cars are less limited to move in space, at certain times of the day travelling by car is still more time-consuming due to traffic congestion [53–55] and difficulties in finding a parking place [56].

Therefore, disregarding an individual’s spatio-temporal travel behavior, the temporality of both transport systems and grocery services and interconnection between these components in accessibility modeling would result in significant overestimation of an individual’s actual access to food stores and underestimation of socio-spatial inequalities. Given the latter, this questions the conventional spatial definition of the “food desert” in health studies, which was well formulated by Widener and Shannon ([38], p. 3)—“[the question is] not just where we might find food deserts, but when”.

### Realistic multimodal accessibility modeling

Another aspect to consider in line with the developments in Geographic Information Systems (GIS) [57] is the advancement in modeling the accessibility by private car and the incorporation of public transport. More realistic accessibility by private car is produced by implementing the stages of a journey and additional components of travel, such as traffic congestions, intersection delays, searching for free parking places, and mandatory walking from/to one’s car [53, 54, 56, 58]. Transport modes other than private car are often ignored and only recently are being incorporated, although with oversimplified assumptions.

In particular, when it comes to public transport with predefined routes and schedules, rather coarse and naïve (constant) assumptions are applied regarding travel distances of routes, travel speeds and transfer waiting times [59–61]. Fortunately, open data such as General Transit Feed Specification (GTFS) and crowdsourcing initiatives such as Open Street Map (OSM) enable advanced public transport and multimodal accessibility modeling. This provides more realistic comparisons of food accessibility between different transport modes, especially in densely populated urban areas with well-established public transport systems [55, 62–64].

In food accessibility research, studies by Farber et al. [47] and Widener et al. [25] are some of the first attempts to implement more advanced public transport modeling for access to food stores. However, many of the comparisons of modal accessibility disparities to date are based on rather oversimplified measures from the shortest travel distances based on road networks [65] to the optimistic “free float” travel times derived from speed limits of road segments [14]. These approaches question the realism and feasibility of such multimodal comparisons in practice. To the authors’ knowledge, this study is the first attempt to implement sophisticated modal accessibility disparity comparisons in health-related research.

We propose that the inclusion of transport modes and an adequate and realistic transport mode comparison are essential to examine food accessibility, given that: (1) from a public health perspective, the use of non-private car usage is related to more active physical activity that is associated with better health outcomes of people [66], whereas (2) from a social justice perspective, certain social groups (youth, elderly, disabled, marginalized or environmentally aware) may not (wish to) have access to a private car and therefore rely on other modes to reach services such as grocery stores [52, 61].

## Methods

### Study design

**Fig. 1:**
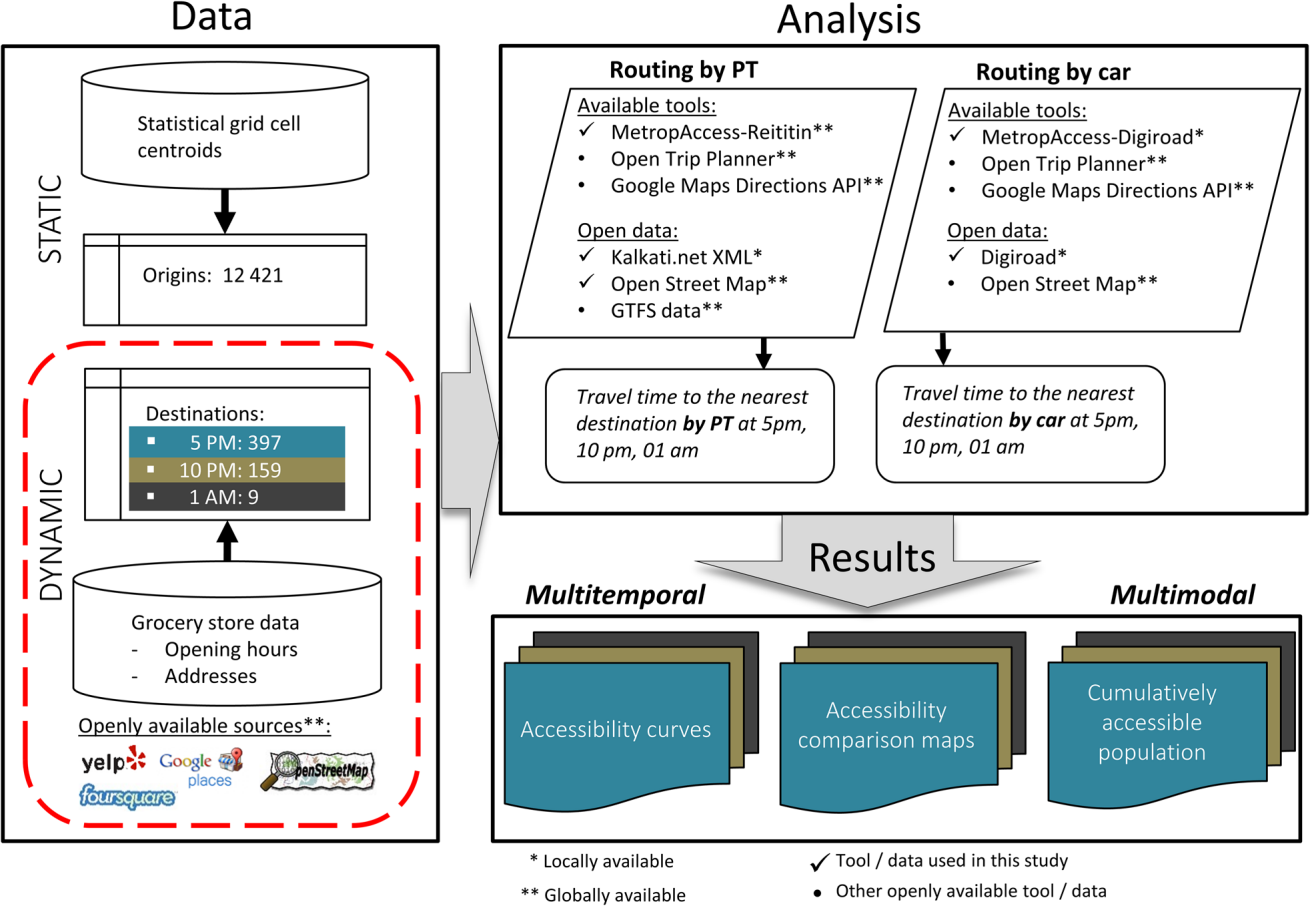
Study design using a nationally/internationally applicable framework to analyze multimodal spatio-temporal accessibility using open data and tools

We explore multimodal, spatio-temporal food accessibility by measuring the travel times by private car and public transportation (PT) between the inhabitants and their closest open grocery store that offers healthy food at different times of the day. We use a widely applicable and sophisticated approach to analyze accessibility, which is presented in Fig. 1.

**Fig. 2:**
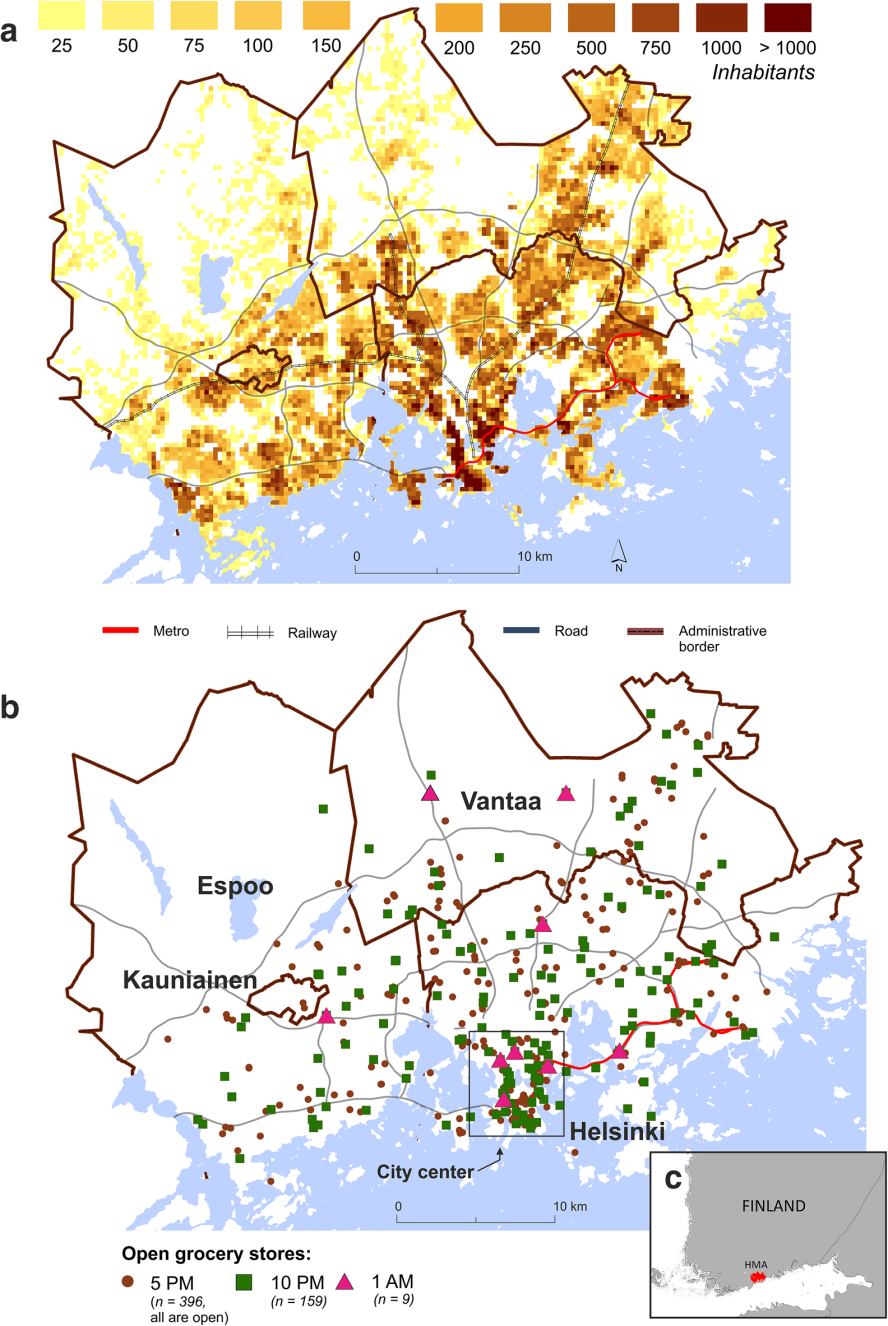
**a** Population density in HMA, **b** spatial distribution of grocery stores at different times of the day, **c** location of the study area in southern Finland

We use three different scenarios to demonstrate how changing service and transport networks in time affect access to healthy food. We chose the times for different scenarios (5 p.m., 10 p.m. and 1 a.m.) based on the general rhythm of the grocery store service network: (1) at 5 p.m. all the stores are open (baseline for the analysis), (2) at 10 p.m. only stores less than 400 m^2^ in size and (3) at 1 a.m. only small 24/7 grocery stores are allowed to stay open, according to legislation. The evolving 24/7 urban societies worldwide increase the demand for grocery shopping beyond non-standard working hours (e.g., at night) for different reasons [46, 67]. Night-time grocery shopping is predominantly due to the limited time availability of people working in shifts or having a variable work schedule. Finland is among the countries having the highest share of night workers (7.5 % in 2014) in Europe [67, 68]. This indicates a potential demand for grocery services around the clock, although there are only few grocery stores in the Helsinki region that are open at night (Fig. 2).

We explore the differences of the scenarios by showing their change in time geographically and in terms of modal differences, and in relation to accessible population by using cumulative accessibility curves and bar plots showing the variation between different municipalities in the Helsinki metropolitan area. In this study, we demonstrate the temporal and modal effects using a register-based night-time population; however, the underlying population could be chosen based on different socio-economic characteristics, for instance, which is often the case in health related research.

### Study area

We use Helsinki metropolitan area (HMA) in Finland as our case study area to demonstrate the effect of temporality and multimodality to health related accessibility analyses. HMA is the capital region of Finland consisting of four municipalities: Helsinki, Espoo, Vantaa, and Kauniainen (Fig. 2). The tiny municipality of Kauniainen is an enclave of Espoo; therefore, we include Kauniainen hereafter as part of Espoo. HMA is the biggest urban region with around one million inhabitants.

HMA is a good example of a region where more sustainable and healthier options for transport (i.e., public transport and cycling) are promoted in government policies and city planning strategies, but where the service centralization and urban sprawl [68] in the last decades have increased the travel need and distances between customers and services, which in turn tends to promote car usage [69–71]. In terms of services, HMA has gone through a structural change where the number of grocery stores has dramatically declined [72] while the average size of the stores has increased [73], mostly concentrating on hypermarkets (>2500 m^2^ in size, selling groceries and general merchandise).

### Data sources

The grocery store data^1^ includes addresses and opening hours of grocery stores that were collected from the websites of store chains or single stores (openly available). We only take into account grocery stores that offer healthy food, thus the product selection must include fruits, vegetables and dairy products (e.g., in supermarkets and hypermarkets, small convenience stores and natural food stores). Ethnic stores, gas stations and kiosks were excluded from the analysis, since their selection of products is typically rather limited. In all, the analysis included 396 stores (see Fig. 2), which were all treated equally (i.e., given equal weight) in the analysis (c.f. Yeager and Gatrell [29]). Other important data sources are listed in Table 1 and described as part of the computational tools.

**Table 1:** Data sources

Data	Phase of analysis	Reference
Grocery store locations and opening hours	Travel time calculation	Grocery store websites
250 m × 250 m statistical grid cells of HMA	Travel time calculation	YKR
Building level population data	Accessibility curves and modal comparisons	SeutuCD’11
MetropAccess-Digiroad (Digiroad)	Travel time calculations by car	MetropAccess
MetropAccess-Reititin (KALKATI & OSM)	Travel time calculation by PT, walking and bicycle	MetropAccess

### Multimodal travel time calculations

We apply 250 m × 250 m statistical grid cells as the most precise spatial units corresponding to the official spatial database of Statistics Finland (i.e., the spatial division of residential population). We calculated the fastest routes from the centroids of all statistical grid cells to the nearest grocery store. Grocery shopping, of course, is influenced by many factors (e.g., accessibility, the selection of products, price level, customer loyalty) [74, 75]; however, to highlight the temporal variability in accessibility we measure travel time only to the (timewise) nearest store without using a gravity-based spatial access model (e.g., Huff’s [76]), which is often used for predicting customer behavior in a more detailed way [77]. Only the open stores at each particular time were included in the calculations.

We applied two freely available open-source tools for spatio-temporal multimodal routing in the Helsinki region to model travel times by private car and public transport. The tools for both travel modes are based on an advanced temporally sensitive door-to-door approach where every stage of a journey between origin and destination is taken into account. This enables the calculation of more realistic and comparable travel times from the origin location to the destination at different times of the day.

In terms of private car use, our accessibility model calculates travel times by taking into account: speed limits and distances of each road segment, cross-road penalties by road class, an average time spent for walking from home to a parking place, an average time spent for searching a vacant parking space at the destination, and an average time spent for walking from a parking place to the destination. For PT, travel times are calculated based on up-to-date PT routes and schedules and also consider walking segments during the trip and transfer times between PT vehicles. Applied models are explained in more detail in Salonen and Toivonen [64] and their technical implementations and source codes are openly available in Github.^2^

We used the MetropAccess-Digiroad^3^ (runs on Windows/Linux with ArcGIS^4^ software) for car travel time/distance calculations that takes into account traffic conditions at different times of the day (rush hour vs daytime). The tool transforms the national road and street database Digiroad^5^ into a network dataset and adds empirically defined intersection delay values to different types of crossings [64, 78]. The tool calculates travel times between given origins and destinations by first defining the “free-flow” drive-through time for each road segment, based on the speed limit information and the length of the respective road segment, then adding an intersection movement delay value specific to the respective road class at certain time, and finally adding an estimate of parking-related time. The intersection movement delay values are derived from floating car measurements where real travel times during different times of the day along different types of roads in HMA are measured with a GPS, similar to Ziliaskopoulos and Mahmassani [53], Vetter and Geisberger [58] and Yiannakoulias et al. [54]. The rush hour intersection delay values were used in the calculation at 5 p.m. and normal daytime values (indicating approximate travel times at noon) at 10 p.m. and 1 a.m.

We used MetropAccess-Reititin^6^ [79] (runs on Windows/Linux, requires Node.js^7^ package) to calculate public transportation travel times and distances between given origin and destination points by public transport based on the advanced door-to-door approach [64]. A modified version of Dijkstra’s algorithm [79, 80] is used to optimize routes between the given origins and destinations. OpenStreetMap^8^ (OSM) is used for calculating the walking parts of the routes, whereas several routing parameters, such as walking speed, departure/arrival date and time, transport mode, and maximum walking distance can be adjusted flexibly. Calculations were based on schedules of a normal weekday (May 5, 2014), walking speed was set to 70 m/min (4.2 km/h), and arrival times were set as 5 p.m., 10 p.m., and 1 a.m. The calculations are based on openly available PT route and schedule data^9^ from the Helsinki Region Transport. As a result, the tool creates a text file where each row presents one origin–destination pair and the calculated route between the respective points, with extensive attribute information (e.g., route travel time, route distance and the travel modes used, access/egress times, etc.).

For short travel distances, walking is often the fastest travel mode and in such case the tool uses only walking for calculating the travel time as it is the most optimal transport mode for reaching the destination. Thus PT in our approach represents either public transport (including walking stages) or just walking.

## Results

### Access to healthy food is affected by temporality

**Fig. 3:**
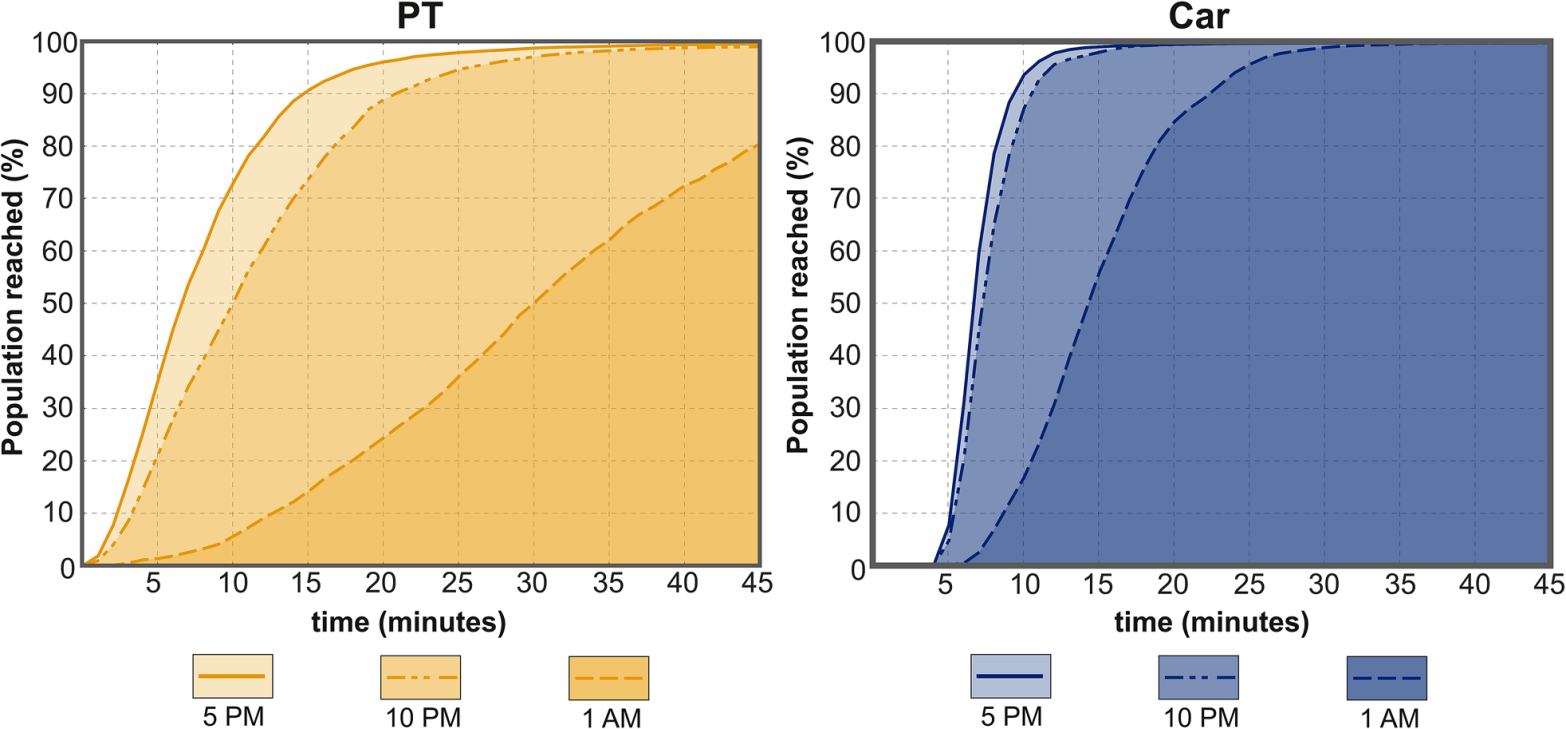
Temporal variation in accessibility to closest grocery stores reached by HMA citizens with different modes of transportation. 100 % ≈ 1,000,000 inhabitants

Our results show that both the transport mode and the time of the day have a prominent impact on accessibility of grocery stores providing healthy food. Figure 3 shows the temporal variation in terms of how fast the population can reach the closest grocery store in our case study region. Depending on the time of the analysis the results are notably different. For instance, if looking at the proportion of population that reaches the closest grocery store within 15 min, the results by PT vary from 90 % (5 p.m.), 70 % (10 p.m.) to only 15 % (1 a.m.). Travel times by private car are significantly lower, thus almost 100 % of the citizens would reach the closest grocery store in 15 min at 5 p.m. and 10 p.m., but only 55 % during the night-time (1 a.m.). Overall, there is significant variation in the results that is caused by temporal changes in the transport system and in the service network.

### Mode of transport affects the spatio-temporal access to healthy food

**Fig. 4:**
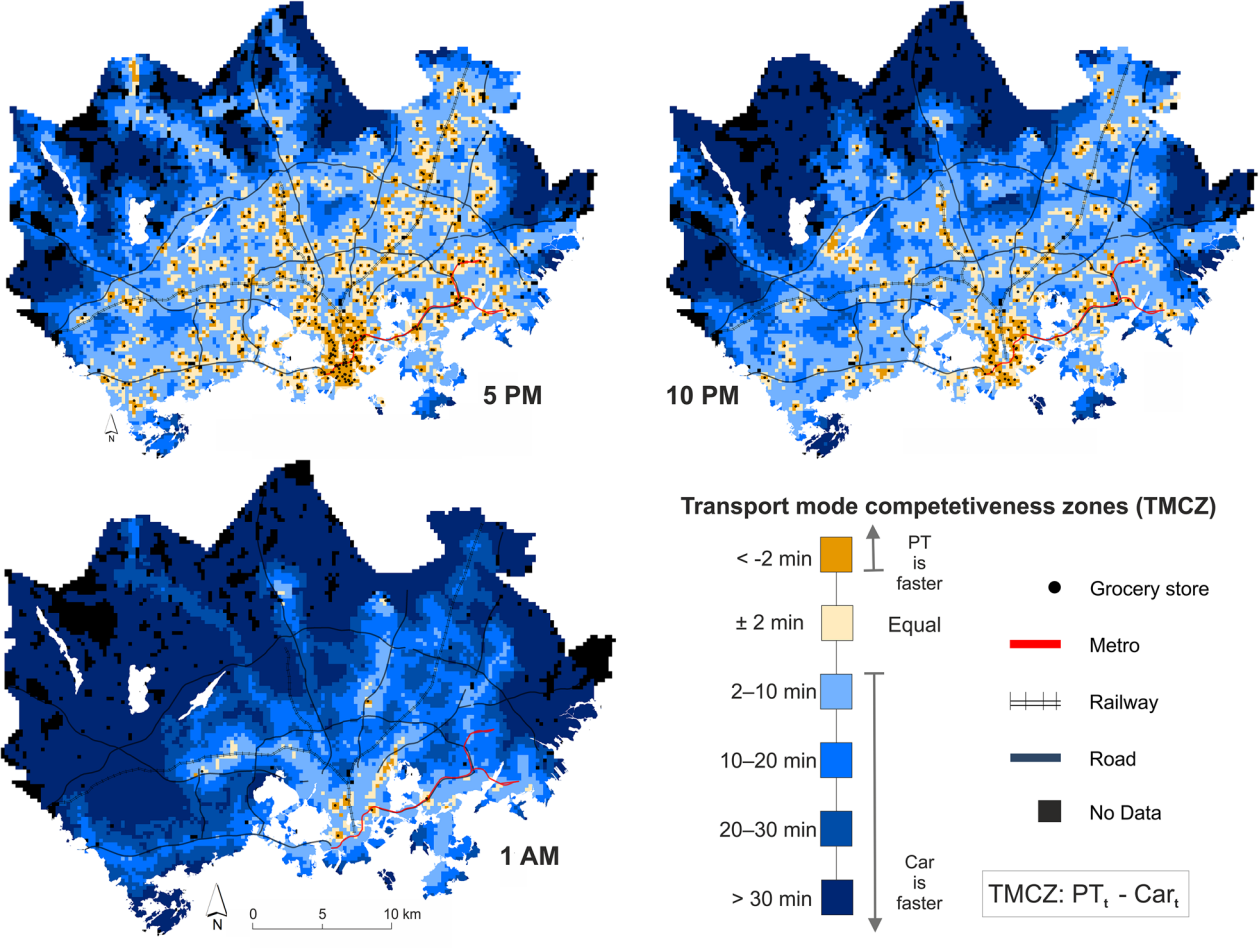
Spatio-temporal accessibility comparisons showing the competitiveness of a transport mode (PT or private car) when travelling from home to the closest grocery store offering healthy food. Values represent the difference in travel times between PT and private car (PT_t_ − Car_t_)

Maps in Fig. 4 extend the previous findings and show the modal differences in accessibility to grocery stores in spatial terms. Results show that there are distinct differences between travel modes in addition to temporal variation. In terms of travel time, PT is actually the fastest travel mode, especially in central Helsinki where PT is always faster (orange areas) or equally fast (white areas) compared to private cars. This might be a slightly surprising finding, although a rational one, as walking and PT are often faster in downtown areas where there is a lot of traffic and it is difficult to find a parking place for a car. In general, even though there are differences between travel modes in terms of travel time, they remain less than 10 min in all areas where there is population (c.f. Fig. 2) at 5 p.m. and 10 p.m. This indicates that PT and private car are equally lucrative options for accessing healthy food in the HMA in terms of travel time, which is a positive result from the perspective of social justice. In outer areas of the HMA private car is still the most viable travel mode in terms of travel time; however, such areas are typically sparsely populated.

**Fig. 5:**
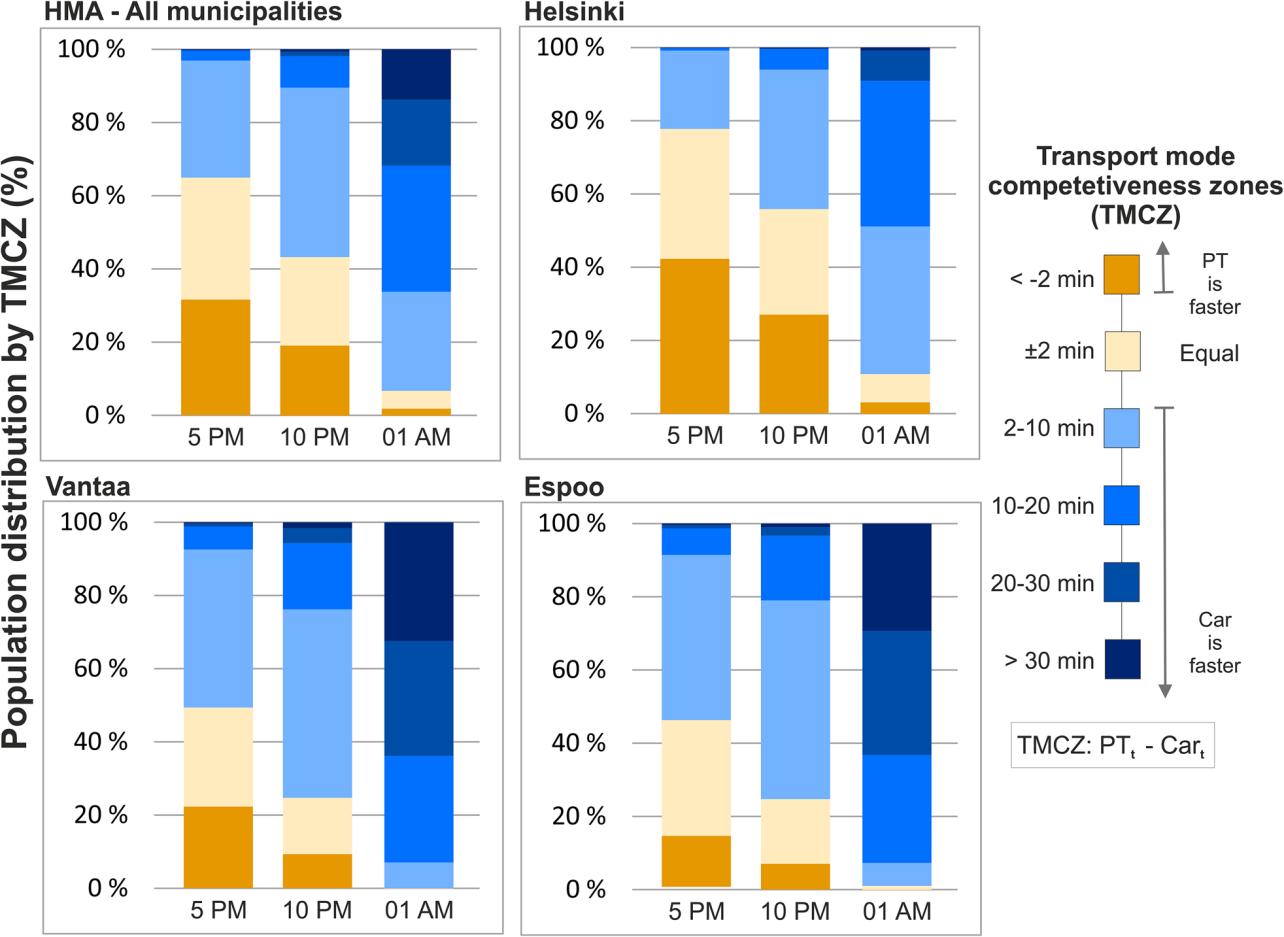
Population distribution by transport mode competitiveness zones (see Fig. 4 for the zones on a map), municipality and time of the day

In health research the spatio-temporal accessibility patterns shown previously are not necessarily the most interesting results, per se. However, when adding underlying population information to the analyses, it is possible to obtain highly relevant and important information supporting health related decision making. Comparing the share of population by transport mode competitiveness zones (Fig. 5) in different municipalities of the HMA at different times of the day shows that there are significant changes in the results, both in temporal and modal terms. Looking at the general situation in the whole HMA, the results show that PT is highly competitive against car use at 5 p.m. when all the food stores are open. At 5 p.m. every third inhabitant lives in areas where PT is faster than a car, and over 60 % of the population lives in areas where PT is a faster or equally fast option compared with using a car. At 10 p.m., when some 60 % of the studied food stores are closed, PT still remains a lucrative travel mode for shopping for more than 40 % of the people. However, at night-time (1 a.m.) the feasibility of PT decreases dramatically and using a car is faster, except in the neighborhoods around the 24 h grocery stores. Hence, car is the faster transport option for 91 % of the people at this given time.

In addition, there are clear differences in competitiveness of PT against car usage between different municipalities of the HMA. In Helsinki, PT is clearly the most viable travel mode for reaching the closest grocery store at 5 p.m.: more than 40 % of the population can access the closest store with PT faster than by car, and nearly 80 % of the population faster or equally fast as by car. In contrast, the situation in Vantaa and Espoo is fairly different as the competitiveness of PT is clearly lower: approximately 20 % of the population can reach the closest store faster by PT in Vantaa and Espoo, and less than half of the population faster or equally fast as by driving with car. Thus, the results support our current understanding that Helsinki is the most PT oriented city in the HMA, whereas Espoo and Vantaa are more car oriented cities.

## Discussion

In this paper we have demonstrated why and how both temporality and multimodality should be integrated to health related studies that include an accessibility perspective. Today, the majority of accessibility related studies in health research apply only static and very simplistic accessibility measures and analyze only a single transport mode. Hence, these studies neglect the multifaceted spatialities of people that daily social life presuppose in our mobile 24/7 societies [36].

The proposed advanced accessibility approach utilizes door-to-door modeling with temporally sensitive accessibility measures. Such an approach was previously difficult to implement in practice due to the lack of reliable data. The recent open data development and novel data sources, such as those listed in Fig. 1 have made it possible to carry out more realistic and up-to-date analyses. Our proposed approach is generic and widely applicable given that it is built on openly available input data sources that are available for hundreds of regions worldwide. For example, we could have used up-to-date spatio-temporal information on public transportation in GTFS^10^ format, which would allow us to make comparative studies, for instance between different European cities in a similar manner to that demonstrated here by comparing the results between municipalities of the HMA. Furthermore, similar to the applied tools in this study [64], other openly available GIS-based accessibility tools, such as Open Trip Planner^11^ and Google Maps Directions API^12^ are freely available for everyone and these can be used to analyze spatio-temporal accessibility patterns by various travel modes.

Our results show that both time and mode of transport clearly affect the outcomes of the analyses and depending on the time and mode of transportation, the understanding of accessibility realities of a city may be very different. In terms of time, the variation in accessibility patterns is evident especially considering PT, but also with private cars, particularly after more significant changes in the service network. In the case of comparing transport modes, our results show that car is not always the fastest travel mode for accessing services: PT can be as fast as, or even faster than private car especially if the PT system is efficient and the service network (e.g., healthy food stores) is fairly dense. These components, in turn, are largely affected by the time of day due to varying opening hours, changing traffic conditions and PT schedules. In our study area, the differences between travel modes were actually fairly modest due to the efficient and comprehensive PT network. However, if similar comparisons were to be made in a region with a less efficient PT network, the modal inequality among residents (or subgroups) could be much higher and accessibility realities might even be contradictory [81]. In this study the analyses were based on static (night time) population distribution; however, novel data sources such as mobile phone data [42] or social media data [82] could be used to evaluate the whereabouts of the population at different times of the day. Incorporating spatio-temporal information about population distribution would further enhance our approach because all analysis components (transport network, service network, and underlying population) would be dynamic.

Why are aforementioned findings significant in terms of health research? In health geography studying how specific groups of people (such as elderly, young people, obese, low-income etc.) can access services such as healthcare or food stores providing healthy food is an increasingly relevant topic. Our results also contribute to the food desert discussion by clearly indicating that time is an essential determinant in access to healthy food (and other health related services), especially for those who work during a non-standard working hours [67]. Night-shift working increases the risk of unhealthier lifestyles, which may be due to poorer access to health promoting services [83, 84]. Moreover, our results show clearly how access to services also depends on transport mode. For example the current spatial concept of “food desert”, referring to a geographical area where disadvantaged (e.g., low-income) residents are lacking spatial access to affordable and healthy food, should be revised to include not only the time dimension [38] but also different modes of transport in order to obtain more realistic outcomes. For example, while low-income population tend to use PT as their main transport mode for daily mobility, it is relevant to measure potential food deserts based on accessibility by PT and not only by private car. Overall, when planning health related systems or implementing policy interventions for tackling health inequalities, realistic information about multidimensional accessibility is needed to ensure equitable services for people with different socio-economic and demographic backgrounds.

## Conclusions

To conclude, we have demonstrated that the realities of accessibility may be very different at different times of the day and from the perspective of a person who uses a car compared to a person who does not; facts that are often neglected in accessibility related health research. Therefore, we propose that more realistic, temporally sensitive, multimodal approaches for accessibility measurements should be adopted in accessibility research [7, 37, 48], health research [12, 57], and in relation to access to healthy food [38, 39, 47].

## Footnotes

1 www.helsinki.fi/science/accessibility/data/MetropAccess-HMA_GroceryStores.zip.

2 https://github.com/AccessibilityRG.

3 http://blogs.helsinki.fi/accessibility/digiroad-tool/.

4 http://www.arcgis.com/.

5 http://www.digiroad.fi/en_GB/.

6 http://blogs.helsinki.fi/accessibility/reititin/.

7 https://nodejs.org/en/.

8 https://wiki.openstreetmap.org.

9 http://developer.reittiopas.fi/pages/fi/kalkati.net-xml-database-dump.php.

10 https://developers.google.com/transit/gtfs/.

11 http://www.opentripplanner.org/.

12 https://developers.google.com/maps/documentation/directions/.
